# Atypical features of nanophthalmic macula- a spectral domain OCT study

**DOI:** 10.1186/1471-2415-12-12

**Published:** 2012-06-06

**Authors:** Aparna Rao, Tapas Ranjan Padhi, Sananu Jena, Souvik Mandal, Taraprasad Das

**Affiliations:** 1 Head Glaucoma Services, LV Prasad Eye institute, Patia, Bhubaneswar, Orissa, India; 2 Retina services, LV Prasad Eye institute, Patia, Bhubaneswar, Orissa, India

**Keywords:** Nanophthalmos

## Abstract

**Background:**

To report atypical features on Spectral domain optical coherence tomography (SD-OCT) in a case of non-familial pure adult nanophthalmos.

**Case presentation:**

A 39 year old male hyperope was found to have biometric and fundus findings typical of nanophthalmos. The additional atypical features included serous pigment epithelial detachment (PED) in right eye and a cuff of subretinal fluid with underlying yellow deposits along superotemporal arcade in the left eye. Fundus flourescein angiogram showed hyperfluorescence due to window defect, dye pooling due to serous PED in right eye and leak superior to disc in right eye and superotemporally in left eye. Cirrus-SD OCT horizontal line scan passing through the fovea showed extensive inner limiting membrane corrugations causing distorted foveal contour in both eyes. A large juxtafoveal serous PED and a small extrafoval PED were seen with folds in the retinal pigment epithelium (RPE)-choriocapillary layer in the right eye.

**Conclusion:**

Structural disruptions in the RPE-choriocapillary complex in the form of folds or juxtafoveal serous PED and RPE folds can be atypical features of nanophthalmic macula better discerned on high resolution OCT.

## Background

Nanopthalmos typically presents with typical clinical findings in a hyperopic small eye [1-5]. Several posterior segment findings have been described earlier including macular folds, retinal cysts or uveal effusion [6]. We report atypical features of a nanophthalmic macula on high resolution imaging which have not been described earlier.

## Case presentation

A 39 year old male presented to us with complaints of poor vision in both eyes since childhood. He did not use any spectacles till presentation.

On examination, he was orthophoric and his unaided and best corrected visual acuity was FC 1/2 m, 20/70, N18 (+14DS/-2DCx10) and FC2m PR accurate, 20/200, N36 (+14DS/-1.5DCx110^0^) in the right and left eye, respectively. Slit lamp showed shallow anterior chamber, intraocular pressure (IOP) by Goldmann applanation tonometry of 20 mm and 18 mm Hg and closed angles on 4 mirror gonioscopy in both eyes. Lens was clear in both eyes. His axial length (15.3 mm& 15.1 mm), corneal diameter and anterior chamber depth were suggestive of nanophthalmos. Central corneal thickness measured 555 microns and 554 microns in the right and left eye, respectively. Review history did not reveal any family history in siblings.

**Figure 1:**
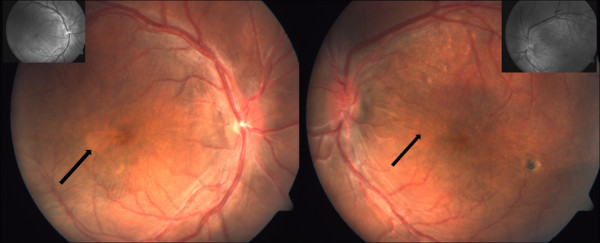
Fundus photograph of the right and left eye with macular striae (black arrow) in adult nanophthalmos (inset showing red free photos).

**Figure 2:**
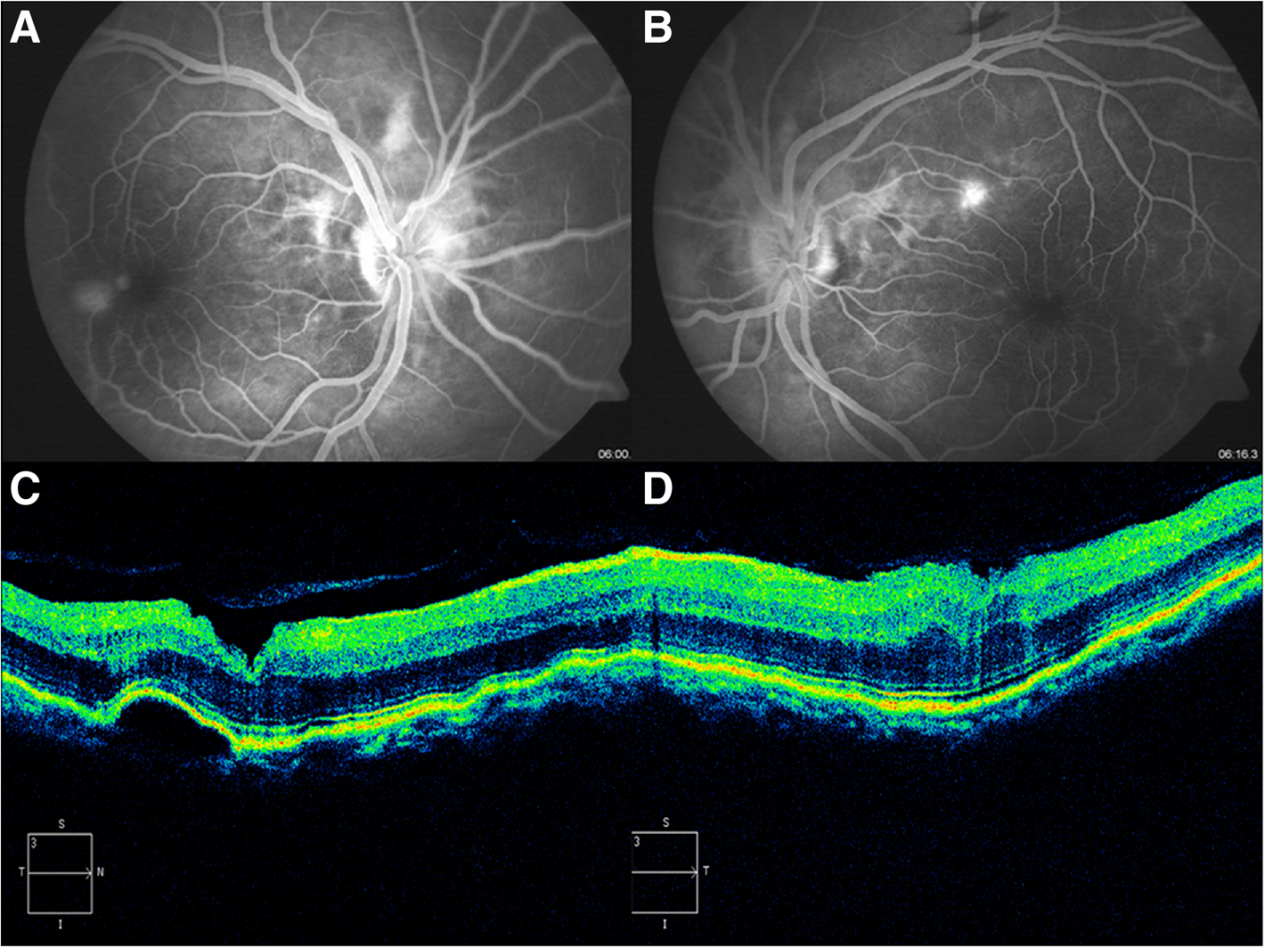
Fundus Flourescein angiography of right eye (A) and Left eye (B) and corresponding Cirrus SD-OCT images (C and D) showing ILM folds in both eyes and juxtafoveal retinal pigment epithelial detachment in the right eye.

On a provisional diagnosis of pure non-familial nanophthalmos, he received prophylactic peripheral laser iridectomy (LPI) in both eyes. Dilated fundus examination showed crowded discs with obliterated cup and dilated engorged non-tortuous vessels in both eyes (Figures 1 &2). There were prominent internal limiting striae radiating from the optic nerve to ½ disc diameter beyond the fovea associated with subretinal deposits in both eyes (Figure 1). There was a cuff of subretinal fluid along the superotemporal arcade with underlying yellow subretinal deposits. There was a pigmented scar in left eye inferotemporal to macula in left eye.

Fundus fluorescein angiography, FFA, revealed hyperfluorescence due to transmission defect at macula in both the eyes,dye pooling in PED in juxtafoveal region in the right eye (Figure 2) and leak along superotemporal arcade in left eye.

Full field flash ERG done showed normal scotopic and photopic response. Humphrey visual fields 24-2 showed peripheral artefacts in both eyes. Cirrus SD-OCT horizontal line scan passing across the fovea showed extensive corrugations involving the inner limiting membrane (ILM) suggestive of ILM striae causing distorted foveal contour in both eyes (Figure 2)*.* A large serous PED was seen encroaching the fovea in the right eye with folds in the RPE-choriocapillary layer. The left eye showed normal vitreoretinal interface and normal intraretinal layer with no folds in the REP-choriocapillary complex.

In view of the above OCT findings, we advised him spectacles with rehabilitative support with low vision devices with advice for periodic follow up.

## Discussion

Nanophthalmic eyes are typically hypermetropic with axial lengths less than 20 mm associated with shallow anterior chamber [1,3,5]. Abnormal deposits of glycosaminoglycans and elevated levels of fibronectin may thicken the sclera causing obstruction of the suprachoroidal drainage pathway and uveal effusion in these cases [6,7].

Papillomacular bands, abnormal thickening of the sclera with glycosaminoglaycans, choroidal congestion, foveal schisis, macular hypoplasia, choroidal thickening, pigmentary retinopathy and uveal effusions have been reported as typical features in posterior microphthamlos or familial nanophthamos [8-10]. In our case of non-familial nanophthalmos, macular striae were associated with RPE involvement in the form of folds and PED in the juxtafoveal area.

Disparity in growth between the sclera and retina probably gives rise to the retinal folds as seen in our patients and reported by others [8]. While amblyopia accounting for reduced vision cannot be ruled out in our case, the macular striae could be responsible for subnormal vision. Such striae could cause photoreceptor dysfunction though this was not the mechanism in our case with normal rod and cone responses. In is unclear from a single case if PED suggests the possibility of progressive changes in the retinal structure with age in an adult nanophthalmic macula.

Pigment cysts, choroidal and non-rhegmatogenous retinal detachments resultant to RPE dysfunction have been reported; [6,8] yet, serous PED as seen in this case has not been reported earlier. While the exact pathogenesis is not known, it is unclear if the PED and subretinal fluid cuff seen in this case represents a focal “effusion” similar to uveal effusion or RPE dysfunction seen in such cases [8]. Nevertheless this case suggests the possibility of progressive changes and structural alterations in nanophthalmic macula with age.

## Conclusion

Structural disruptions in the RPE-choriocapillary layer including PED and RPE folds can be atypical features of nanophthalmic macula better discerned on high resolution SD-OCT.

### Consent

"Written informed consent was obtained from the patient for publication of this Case report and any accompanying images. A copy of the written consent is available for review by the Series Editor of this journal."

## Competing interests

The authors declare that they have no competing interests.

## Authors’ contribution

APR, TRP and TPD have made contributions to conception and design, or acquisition of data, or analysis and interpretation of data; all have been involved in drafting the manuscript or revising it critically for important intellectual content; SJ and SM have help acquire images and collect data. All authors have reviewed and approved of the manuscript.

## Pre-publication history

The pre-publication history for this paper can be accessed here:

http://www.biomedcentral.com/1471-2415/12/12/prepub
